# Intravital imaging of orthotopic and ectopic bone

**DOI:** 10.1186/s41232-020-00135-6

**Published:** 2020-11-02

**Authors:** Kunihiko Hashimoto, Takashi Kaito, Junichi Kikuta, Masaru Ishii

**Affiliations:** 1 Department of Orthopedic Surgery, Graduate School of Medicine, Osaka University, Osaka, 565-0871 Japan; 2 Department of Immunology and Cell Biology, Graduate School of Medicine & Frontier Biosciences, Osaka University, 2-2 Yamada-oka, Suita, Osaka, 565-0871 Japan

**Keywords:** Intravital imaging, Multiphoton microscopy, Regeneration, Bone, Ectopic bone

## Abstract

Bone homeostasis is dynamically regulated by a balance between bone resorption by osteoclasts and bone formation by osteoblasts. Visualizing and evaluating the dynamics of bone cells in vivo remain difficult using conventional technologies, including histomorphometry and imaging analysis. Over the past two decades, multiphoton microscopy, which can penetrate thick specimens, has been utilized in the field of biological imaging. Using this innovative technique, the in vivo dynamic motion of bone metabolism-related cells and their interactions has been revealed. In this review, we summarize previous approaches used for bone imaging and provide an overview of current bone tissue imaging methods using multiphoton excitation microscopy.

## Background

Bone homeostasis is dynamically regulated internally and externally via interactions among osteoblasts, osteoclasts, osteocytes, other organs, and other cell types. Conventional histological evaluation of bone tissue has been performed on histological sections ex vivo; this evaluation is limited to “static” analysis of cell morphology and gene and protein expression. Bone histomorphometric analysis can quantify the rates of bone formation and resorption during a certain period but cannot visualize real-time “dynamic” behaviors, interactions, and functions among osteoblasts, osteoclasts, osteocytes, and other cell types.

Because conventional laser beams used for microscopic observation cannot penetrate the thick mineralized cortical bone, numerous approaches including micro-computed tomography imaging [1–4], Raman microspectroscopy imaging [5, 6], and magnetic resonance imaging [7] have been utilized for indirectly visualizing the inside of the bone through time-lapsed in vivo imaging. However, it remains difficult to perform real-time analysis of bone dynamics.

The advent of multiphoton microscopy has launched a new era in the field of biological imaging of the bone. The longer wavelength light sources used in multiphoton excitation microscopy enable deeper tissue penetration because less scattering occurs, and there is less laser-induced damage to the tissue; the lower levels of photobleaching of the imaged fluorophores by using near-infrared lasers enables longer observation times compared with those of conventional fluorescent microscopy. Using this innovative technique, the dynamic motion of osteoblasts and osteoclasts and their interactions inside skeletal bone have been clarified. We recently established a novel intravital imaging system for ectopic bone formation as a powerful tool for optimizing bone tissue regeneration. In this review, we provide an overview of current bone tissue imaging including skeletal bone and ectopic bone formation using multiphoton excitation microscopy.

### Intravital imaging of skeletal bone with multiphoton excitation microscopy

In vivo imaging can be divided into intravital imaging and tissue explant imaging [8]. In intravital imaging, experimental animals are kept alive under anesthesia, and tissues of interest are exposed for observation. In contrast, in tissue explant imaging, the tissues harvested from experimental animals are maintained under viable conditions for observation in culture media. In this review, we discuss only intravital bone imaging with multiphoton excitation microscopy. Mouse calvarial bone, in which the distance from the bone surface to the bone cavity is only 80–120 μm, was used in studies to visualize the bone-marrow cavity. This is because thick cortical bone consists of crystalized calcium phosphate, which prevents visualization at depths greater than 150–200 μm, even with the use of a near-infrared laser [8]. However, in addition to the calvarial bone, there are reports of imaging using multiphoton excitation microscopy on the long bones such as the femur [9] or tibia [10, 11]. Intravital imaging of the marrow of long bones is more invasive because surgical treatment (bone thinning or drilling) of the cortical bone is required. In addition, for the reduction of artifacts (body motion) during the intravital imaging experiments, the imaging box and the anesthetized mouse are maintained at a constant warm temperature using heated air. Heart rate is monitored using an electrocardiogram monitor device, and the concentration of the anesthetic gas is adjusted by using the heart rate as a guide.

### Visualization of osteoclasts, osteoblasts, and matrix

To visualize osteoclasts and osteoblasts, genetically modified (knock-in) mice expressing a fluorescent protein in cell-specific genes are employed. To visualize mature osteoclasts, transgenic mice expressing fluorescent proteins under the control of the tartrate-resistant acid phosphatase (TRAP) promoter (TRAP-tdTomato mice) or the ATP-driven proton pump (V-type H+-ATPase a3 subunit) promoter (a3-GFP mice) are used [12–15]. To visualize osteoclast precursors, knock-in mice expressing enhanced green fluorescent protein (EGFP) under the promoter of fractalkine receptor (CX3CR1), which is expressed mainly on monocyte-lineage cells, including osteoclast precursors, was used (CX_3_CR1-EGFP mice) [16, 17].

To visualize osteoblasts, transgenic mice expressing GFP or enhanced cyan fluorescent protein (ECFP) under the promoter of type I collagen in osteoblasts (Col 2.3-GFP and Col 2.3-ECFP) is generally used [13, 15, 18–20] (Table 1).

**Table 1:** Fluorescent-labeled mice for intravital multiphoton imaging in bone

Target gene/promoter	Fluorescence (color)	Labeled cells	Report (development)	Report intravital imaging)
CX3CR1	EGFP (green)	Macrophage	Jung et al. Mol. Cell. Biol. (2000) [16].	Ishii et al. Nature (2009) [17].
V-ATPase a3 subunit promoter	GFP (green)	Macrophage	Sun-Wada et al. J Cell Sci. (2009) [14].	Kikuta et al. J. Clin. Invest. (2013) [12].
TRAP (exon 2)	tdTomato (red)	Osteoclasts	Kikuta et al. J. Clin. Invest. (2013) [12].	Kikuta et al. J. Clin. Invest. (2013) [12]. Furuya et al. Nat. Commun. (2018) [13].
Rat COL1A1 promoter (2.3 kb)	ECFP (cyan)	Osteoblasts	Furuya et al. Nat. Commun. (2018) [13].	Furuya et al. Nat. Commun. (2018) [13]. Hashimoto et al. Sci Rep. (2020) [15].
Rat COL1A1 promoter (2.3 kb)	GFP (green)	Osteoblasts	Kalajzic et al. J. Bone Miner. Res. (2002) [18].	Kalajzic et al. J. Bone Miner. Res. (2002) [15]. Villa et al. Tissue Eng Part C Methods. (2013) [19]. Huang C, et al. J Bone Miner Res. (2015). [20]

*CX3CR1* C-X3-C motif chemokine receptor 1, *EGFP* enhanced green fluorescent protein, *V-ATPase* V-type H+-ATPase, *GFP* green fluorescent protein, *TRAP* tartrate-resistant acid phosphatase, *COL1A1* collagen type 1 alpha 1, *ECFP* enhanced cyan fluorescent protein

To further visualize the functional status of cells, chemical fluorescent probes are being developed such as pH-activatable probes, by which a low-pH region created by bone-resorbing osteoclasts can be visualized [12, 13, 21, 22] (Table 2).

**Table 2:** Small molecular fluorescent probes for intravital multiphoton imaging in bone

Small molecular probe	Fluorescence	Cell function	Report (development)	Report (intravital imaging)
BAp-E	Green	Bone resorption (osteoclast)	Kowada et al. J Am Chem Soc. (2011) [21].	Kikuta et al. J. Clin. Invest. (2013) [12].
pHocas-3	Green	Bone resorption (osteoclast)	Maeda et al. Nat. Chem. Biol. (2016) [22].	Furuya et al. Nat. Commun. (2018) [13].

*BAp-E* boron-dipyrromethene-based (BODIPY-based) H+-sensing fluorescent probe, *pHocas-3* pH-activatable fluorescent probe for osteoclast activity sensing-3

By using near-infrared lasers for multiphoton excitation, collagen fibers in the bone matrix can be visualized by a nonlinear optical process named as second harmonic generation (SHG), without additional fluorescent labeling [15, 19, 23–25]. Additionally, administration of alizarin can be performed to visualize calcified bone matrix [15, 19, 26] (Table 3).

**Table 3:** Other methods for bone tissue visualization in intravital multiphoton imaging

Target molecule/protein	Visualization (color)	Target tissue	Report (development)	Report (intravital imaging)
Noncentrosymmetric molecular organization (collagen etc.)	SHG (blue, etc.*)	Bone etc.	Freund et al. Biophys J (1986) [23].	Hashimoto et al. Sci Rep. (2020) [15]. Villa et al. Tissue Eng Part C Methods. (2013) [19].
Ca^2+^	Alizarin (red)	Bone (mineral)	O’Brien et al. J Biomech. (2002) [26].	Hashimoto et al. Sci Rep. (2020) [15]. Villa et al. Tissue Eng Part C Methods. (2013) [19].

*SHG* second harmonic generation

*Color changes based on the wavelength of incidental laser

**Table 4:** Merits and demerits of multiphoton excitation microscopy

Multiphoton excitation microscopy	
Merits	Demerits
1. Time-lapse intravital imaging	1. Visualization of only fluorescently labeled target cells and SHG
2. Imaging without tissue preparation and staining	2. Limitation of penetration depth
3. Visualization of collagen fibers as SHG	3. Difficulty in visualizing inflamed tissues with effusion or tissues with bleeding

Multifluorescent images are acquired via direct detection of fluorescence using four external non-descanned detectors equipped with dichroic and emission filters. The acquired images are subjected to channel unmixing using the NIS-Elements integrated software (Nikon) for autofluorescence and crosstalk reduction [13, 15].

### Dynamic visualization of osteoclasts

Osteoclasts, which have a unique hematopoietic origin and move dynamically among bone cells, can be visualized by intravital imaging, which has provided insights into these cells. Osteoclast precursors circulate in the blood vessel and migrate to the bone surface to perform their bone resorptive activity. However, the mechanism underlying the control of osteoclast precursor migration remains unclear. We demonstrated that sphingosine-1-phosphate (S1P), an abundant lipid mediator in the blood, regulates the migration of osteoclast precursors between blood vessels and the bone surface through two S1P receptors (S1PR1 and S1PR2) expressed on osteoclast precursors [17]. The action mechanism of the anti-osteoporosis agent active vitamin D was shown to involve reduced expression of S1PR2 on osteoclast precursors at the bone surface and subsequent migration of osteoclast precursors from the bone surface to the blood vessels [27].

Combined visualization and quantification of osteoclast motility and the acidic environment created by osteoclasts using pH-sensing chemical fluorescent probes clarified that mature osteoclasts can be classified into two different types based on their motility and function, that is, the static-bone resorptive (R-type) and moving-non-resorptive (N-type) [12]. Intravital imaging enabled real-time evaluation of the conversion from N-type to R-type osteoclasts through direct cell-to-cell contact with Th17 cells (a subset of RANKL-expressing CD4+ T cells) and intravenous administration of RANKL to induce the formation of mature osteoclasts.

### Dynamic interaction between osteoclasts and osteoblasts

The coupling of bone formation and resorption has been increasingly recognized as a complex, dynamic process. Cell-to-cell contacts between osteoblasts and osteoclasts play an important role in coupling [28], but the spatiotemporal relationship and functional changes caused by the contacts remain unclear. We recently performed intravital imaging of the interaction between osteoblasts and osteoclasts by using double transgenic mice expressing EGFP driven by the type I collagen promoter in osteoblasts and tdTomato under control of the TRAP promoter in osteoclasts [13]. We demonstrated that osteoclasts do not perform bone resorption when they are in contact with osteoblasts and engage in bone resorptive activity when they are not in contact with osteoblasts. These results suggest that osteoblasts control the bone resorption activity of osteoclasts via direct contact.

### Intravital imaging of ectopic bone formation

**Fig. 1:**
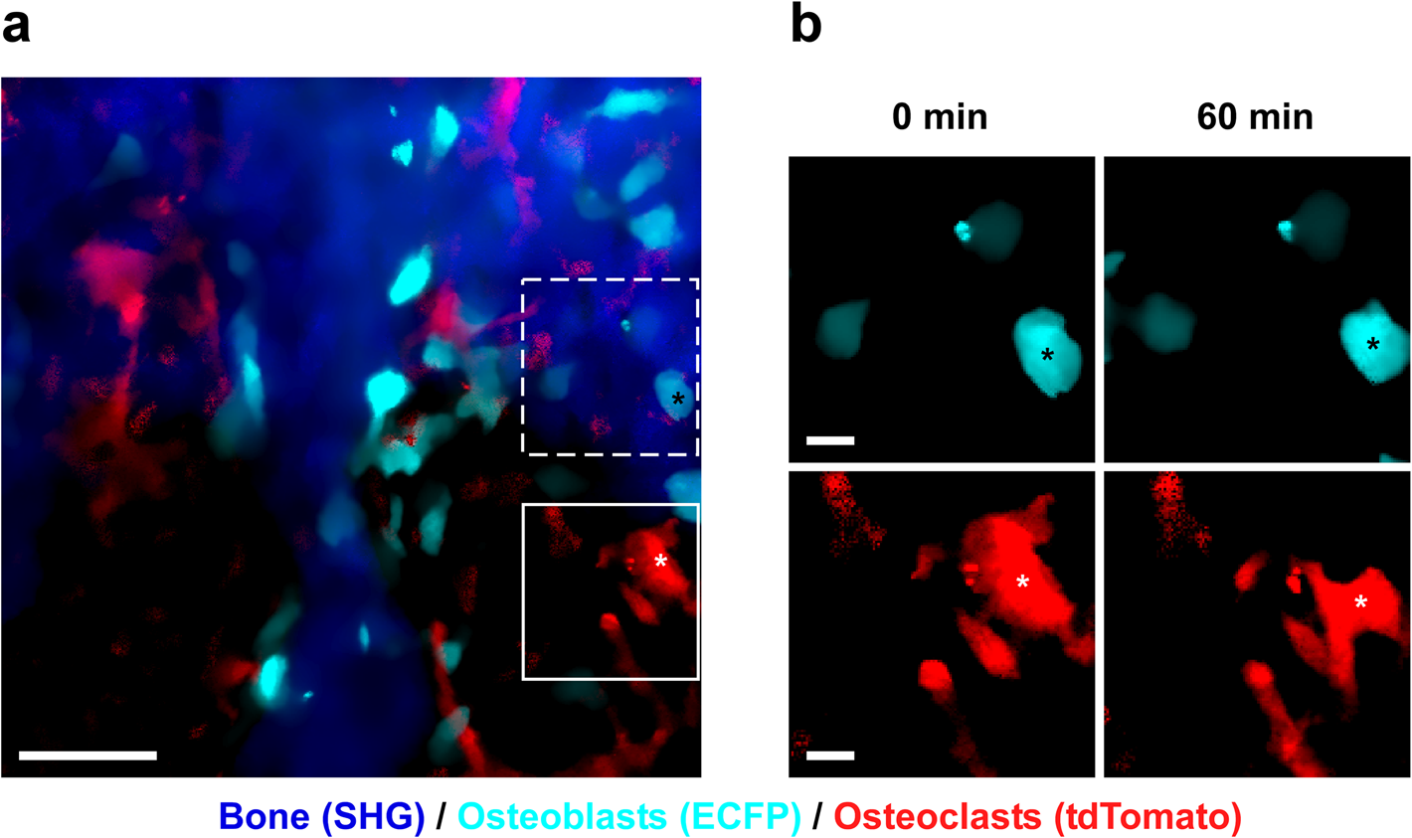
Intravital two-photon microscopy images of calvarial bone in Col2.3-ECFP/TRAP tdTomato mice. **a** Representative image of the bone cavity at calvarial bone. Cyan, osteoblasts expressing Col2.3-ECFP; red, osteoclasts expressing TRAP tdTomato; blue, bone tissues (second harmonic generation, SHG). Scale bar, 50 μm. **b** Magnified images of osteoblasts (region delineated by dotted lines in **a**) and osteoclasts (region outlined in **a**) captured at an interval of 60 min. Although the osteoblasts underwent very few morphological changes, the osteoclasts underwent dramatical changes in morphology. Scale bar, 10 μm

**Fig. 2:**
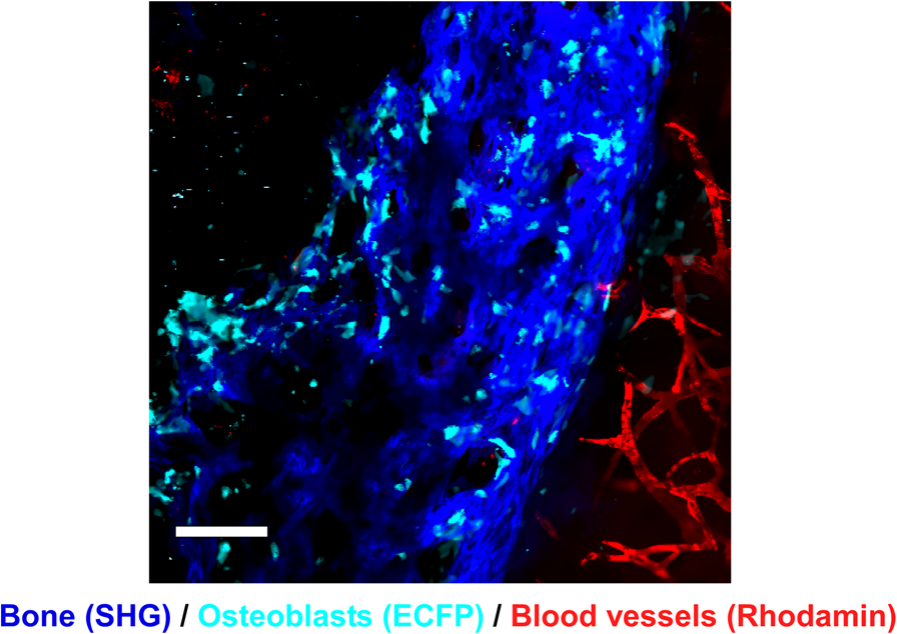
Representative intravital two-photon microscopy images of ectopic bone formation area in Col2.3-ECFP mice (visualization of blood vessels). Visualization of osteoblasts, newly formed bone (collagen), and blood vessels after implantation of a collagen sponge containing BMP-2 [15]. Cyan, osteoblasts expressing Col2.3-ECFP; red, blood vessels stained with rhodamine; blue, collagen fibers (second harmonic generation, SHG). Scale bar, 100 μm

**Fig. 3:**
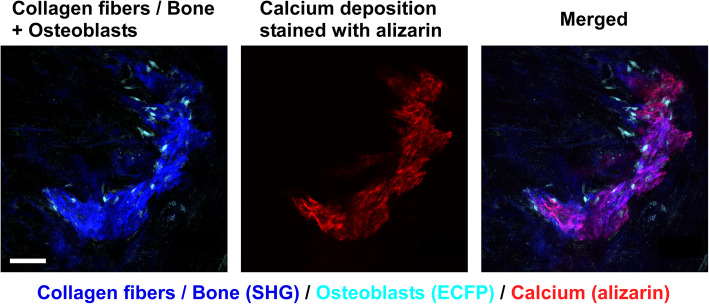
Representative intravital two-photon microscopy images of ectopic bone formation area in Col2.3-ECFP mice (visualization of calcium deposition). Visualization of osteoblasts, newly formed bone (collagen), and calcium deposition stained with alizarin after implantation of a collagen sponge containing BMP-2 [15]. Cyan, osteoblasts expressing Col2.3-ECFP; red, calcium deposition stained with alizarin; blue, collagen fibers (second harmonic generation, SHG). Scale bar, 100 μm

**Fig. 4:**
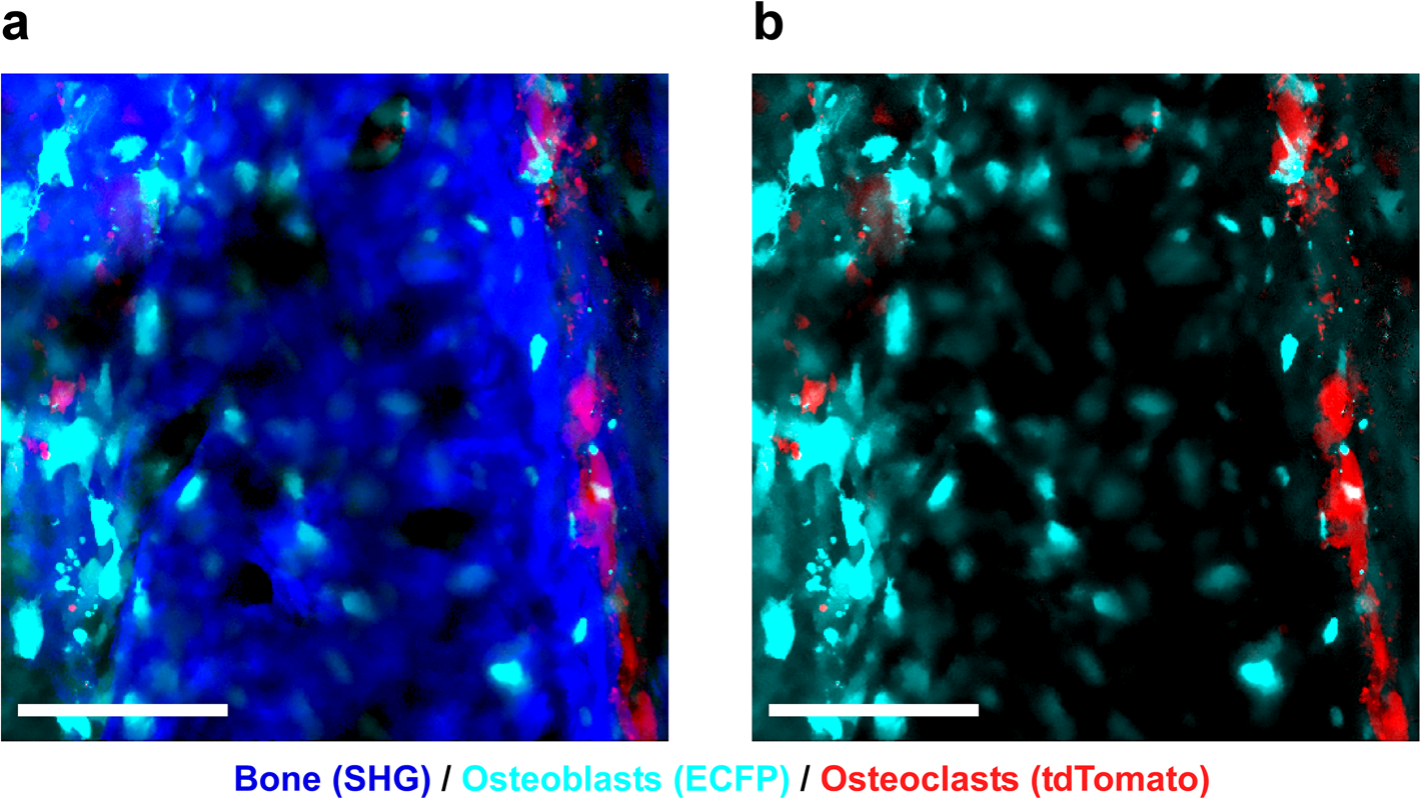
Representative intravital two-photon microscopy images of ectopic bone formation area in Col2.3-ECFP/TRAP tdTomato mice. Visualization of osteoblasts, osteoclasts, and newly formed bone (collagen) after implantation of a collagen sponge containing BMP-2 [15]. Cyan, osteoblasts expressing Col2.3-ECFP; red, osteoclasts expressing TRAP-tdTomato; blue, collagen fibers (second harmonic generation, SHG). Scale bar, 100 μm

**Fig. 5:**
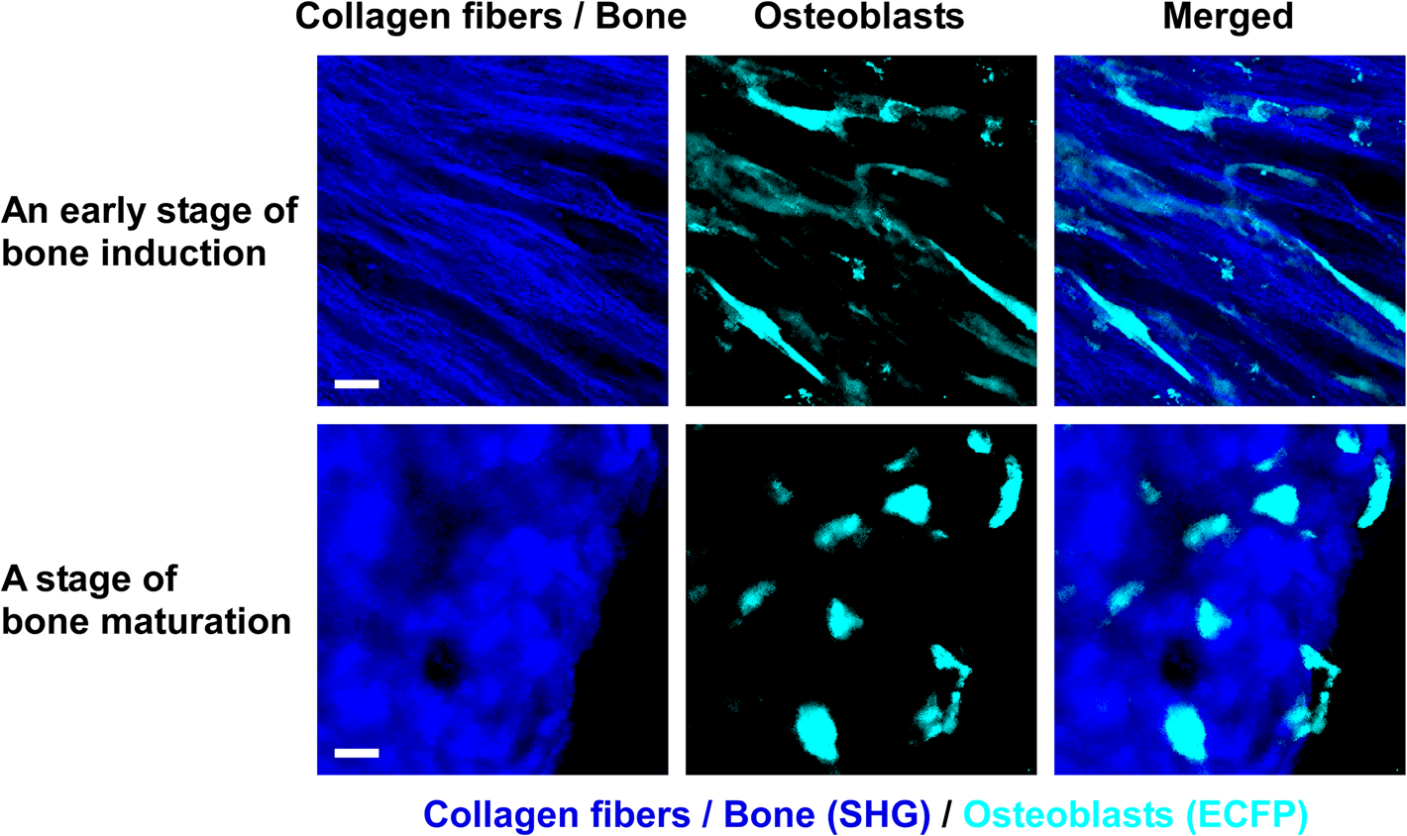
Visualization of osteoblasts and collagen fibers (bone) during the bone induction process. At an early stage of bone induction, the morphology of osteoblasts shows spindle shape and collagen fibers are arranged in a specific (anisotropic) direction (upper panels). As the bone matures, the appearance of collagen fibers becomes isotropic. The osteoblasts spread across the surface of the induced bone, and the morphology of osteoblasts changes into a round or cuboidal shape [15]. Cyan, osteoblasts expressing Col2.3-ECFP; blue, bone tissues (second harmonic generation, SHG). Scale bar, 20 μm

Intravital imaging of skeletal bone by multiphoton excitation microscopy has revealed the in vivo dynamic motion of osteoclasts and osteoblasts, as well as their interactions with each other (Fig. 1). This innovative technique may also be applicable in the field of bone regeneration. To optimize bone regeneration, the process by which new bone is created, it is important to understand the temporospatial appearance and motility of osteoclasts and osteoblasts and the bone formation process. Indeed, the investigations on BMSC-mediated calvarial bone defect repair using multiphoton excitation microscopy have been reported [19, 20]. In the field of bone tissue engineering, bone morphogenetic protein (BMP) plays a central role owing to its potent bone induction ability [29]. However, the dose-dependent inflammation-related side effects of BMP prevent its widespread use [30, 31]. One possible reason for the difficulty in optimizing BMP-induced bone formation is the limited understanding of this process in vivo. We successfully established a method for intravital imaging of the BMP-2-Induced ectopic bone formation process [15]. The temporospatial dynamic bone formation process, which is initiated by angiogenesis, the recruitment of osteoblasts and osteoclasts, the formation of collagen fibers, and deposition of bone minerals were visualized by intravital imaging of Col2.3-ECFP mice and Col2.3-ECFP/TRAP tdTomato mice (Figs. 2, 3, and 4). Additionally, we detected spindle-shaped osteoblasts and unidirectional collagen fibers, with the osteoblasts and collagen fibers showing an orientational correlation in the early stage of ectopic bone formation (Fig. 5).

Furthermore, it is known that intermittent administration of teriparatide accelerates osteogenic differentiation of mesenchymal progenitor cells instead of adipogenic differentiation [32], reactivates lining cells [33], and suppresses apoptosis in osteoblasts [34]; it also activates bone remodeling, which results in the increased bone resorptive activity of osteoclasts. Moreover, in this study, intermittent administration of teriparatide was shown to increase BMP-induced bone volume with the increase in the number of osteoblasts, and caused a decrease in the dynamic morphological changes (activated resorptive activity) in osteoclasts.

Intravital imaging can be used to visualize the whole BMP-2-induced bone induction process, contributing to the understanding of ectopic bone formation and providing a foundation for optimizing this process.

### Future perspective

Intravital imaging has the greatest advantage with regard to spatiotemporal visualization of living tissues, which cannot be achieved using other methods. However, current imaging techniques have several limitations (Table 4). First, not all objects can be observed in the visual fields of multiphoton microscopy. Because only fluorescent labeling and SHG allow us to see the target cells or organs, we should not misunderstand the lack of a signal as showing an open field. As diverse structures and cellular components are probably present, observations need to be interpreted carefully. Second, the penetration depth in two-photon microscopy is up to 800–1000 μm in soft tissues and 200 μm in hard tissues (e.g., bone). Therefore, there are only limited areas where this imaging can be applied. However, the recently developed three-photon microscopy substantially improves the penetration depth and is expected to overcome these limitations with its resolution [35]. Moreover, because of the wide scattering of light, it is difficult to visualize inflamed tissues with effusion or tissues with bleeding using multiphoton microscopy. To resolve these problems, technical innovations in optical systems and fluorophore are expected in the future.

## Conclusions

Intravital imaging has made it possible to visualize the “real-time” dynamics of living cells and biological phenomena caused by cell-to-cell interactions and interventions in various organs. Application of this technique to bone imaging in the past 10 years has provided insights into bone-related processes. Rapid advances in the development of optical instruments and functional chemical fluorescent probes [9] will enable visualization of the dynamics and function in bone.

## Data Availability

Not applicable.

## Abbreviations

TRAP: Tartrate-resistant acid phosphatase
EGFP: Enhanced green fluorescent protein
ECFP: Enhanced cyan fluorescent protein
SHG: Second harmonic generation
S1P: Sphingosine-1-phosphate
BMP: Bone morphogenetic protein
Col 2.3: Promoter of type I collagen in osteoblasts
